# Emotion recognition based on feature weight analysis of multiple physiological signals

**DOI:** 10.1371/journal.pone.0345184

**Published:** 2026-03-31

**Authors:** Qi Li, Yunqing Liu, Fei Yan

**Affiliations:** 1 Department of Electronics and Information Engineering, Changchun University of Science and Technology, Changchun, China; 2 Jilin Provincial Science and Technology Innovation Center of Intelligent Perception and Information Processing, Changchun, China; National University of Sciences and Technology, PAKISTAN

## Abstract

Emotion recognition stands as a complex and prominent challenge within contemporary artificial intelligence research. Deep learning on physiological signals has boosted emotion recognition, yet unimodal limits, ignored channel importance, and temporal cues hinder feature extraction. Within this investigation, we introduce a multimodal framework for emotion recognition, integrating various attention mechanisms to refine feature extraction from multimodal physiological data, which in turn elevates the precision of emotion detection. Firstly, this paper fully exploits the distributed nature of multi-channel EEG signals by extracting micro-differential-entropy (DE) emotion matrices from both EEG and peripheral physiological signals. A channel-attention mechanism is then introduced to measure the similarity among electrode-channel samples of the physiological signals, yielding sample-importance weights that are subsequently probabilistically redistributed across the channels. With these reweighted signals, depthwise-separable convolutional neural networks and long short-term memory networks are employed to capture their spatial and channel-attention information. Secondly, recognizing that latent emotional information exists between temporal slices of multimodal physiological signals, the paper fuses the extracted features from different modalities into a unified representation. A multi-head attention mechanism is integrated into a recurrent network with ordered neurons to explore the relative importance of temporal sequences across physiological samples, thereby achieving emotion recognition. Finally, the proposed approach is evaluated on two distinct datasets, and experimental results demonstrate its strong generalization capability.

## 1. Introduction

Emotion recognition, a fundamental application of artificial intelligence, is extensively applied in medical care, education, criminal investigation, service robots, and more [1–2]. Past studies mainly relied on facial expressions and speech signals for tasks related to emotion recognition [3]. However, these data are susceptible to subjective human control and have a low level of credibility, whereas emotion detection based on physiological electrical signals overcomes this drawback and has better credibility. In addition, different physiological signals carry richer emotional information and provide an important clue for more accurate recognition of human emotions. Currently, EEG measurements have been widely adopted in affective computing research owing to advantages such as exceptional temporal resolution, non-invasive operation, and instantaneous discriminative capacity [4].

Recent advancements in deep learning have found widespread application across diverse domains including visual computing systems [5], linguistic data processing frameworks [6], and biomedical signal interpretation techniques [7], outperforming conventional machine learning approaches in predictive accuracy and pattern recognition capabilities. Within the specialized field of emotion recognition through EEG signal analysis, various deep neural architectures have been successfully adapted. Alhagry's research team [8] implemented a recurrent neural network with long short-term memory (LSTM) units for emotion classification using neurophysiological data, attaining 87.99% classification accuracy on standardized emotion recognition benchmarks through temporal feature extraction from raw EEG signals.

Within the DEAP dataset framework, researchers have addressed individual variability in EEG patterns through innovative approaches. Li et al. [9] developed a Depth-Adaptive Network (DAN) that improves cross-subject feature transfer by dynamically adjusting network architecture depth. Their methodology was validated through emotion classification experiments utilizing SEED and SEED-IV datasets, showcasing robust performance in multi-participant affective state identification. Advancing the domain, Zheng's team [10] proposed a CNNFF neural architecture specifically designed to extract

The study explores interconnections between electrode channels in three-dimensional feature maps to improve emotion detection accuracy. Experimental evaluations conducted on the DEAP dataset demonstrated average classification accuracies reaching 94.04% for arousal detection and 93.61% for valence assessment. Researchers Gao et al. [11] developed an innovative emotion analysis framework combining multi-level convolutional neural architecture (MCNN) with differential entropy measurements and neural connectivity patterns. This methodology yielded enhanced performance metrics through synergistic integration of spatial-temporal brain signal characteristics and deep learning mechanisms.

Investigations conducted using the SEED database yielded an experimental accuracy of 91.45%. Through the implementation of a three-dimensional convolutional neural network architecture incorporating multi-scale kernel dimensions by Su et al. [12], researchers attained 95.67% accuracy in four-category classification tasks when evaluating the DEAP dataset.

However, relying solely on EEG measurements presents limitations in comprehensively assessing emotional states. Incorporating multimodal physiological data – including EMG recordings, electrodermal activity, and cardiac electrical patterns captured concurrently – has demonstrated improved reliability in emotion-related data interpretation. Bagherzadeh et al. [13] introduced a Parallel Stacked Autoencoder (PSAE) approach, which segments EEG data alongside eight supplementary physiological metrics from the DEAP database into distinct processing channels.

The study partitions 12 feature subsets and processes these through a series of parallel stacked autoencoders, achieving four-category classification of emotional valence and activation levels with a mean accuracy reaching 93.6%. In related research, Huang et al. [14] introduced an ensemble convolutional neural network (ECNN) framework to identify four mental states – calmness, distress, physiological arousal, and anxiety – by leveraging multimodal physiological data encompassing electroencephalographic (EEG) recordings, skin conductance measurements, breathing patterns, and ocular electrical signals.

When evaluated using the DEAP dataset, the framework attained a mean classification precision of 82.92%. Ma et al. [15] proposed a hybrid architecture merging residual network-derived spatial skip links with LSTM-based temporal pathways. This design facilitates effective extraction of hierarchical features encoding affective characteristics to support emotion categorization tasks. Empirical validation on the DEAP benchmark demonstrated valence prediction accuracies reaching 92.3%.

The recognition accuracy reached 92.87% for arousal in related studies. Zheng et al. [16] developed a comprehensive multimodal framework for emotion detection that combines six paired temporal EEG channels (including FT7, T7, TP7 and their right-hemisphere counterparts FT8, T8, TP8) with ocular movement data. The methodology commences with the integration of fundamental characteristics from both neural electrical activity and visual tracking signals. Following this initial combination, a Bimodal Deep Auto-Encoder (BDAE) architecture is utilized to derive more sophisticated latent representations from the dual-modality inputs. In complementary research, Liu et al. [17] implemented deep canonical correlation analysis (DCCA) to investigate emotional states through multimodal.

Rayatdoost and colleagues [18] developed a cross-modal encoder framework designed to simultaneously capture features from neural and physiological signals including EEG, EMG, and EOG. The methodology begins by converting power spectral density (PSD) characteristics from distinct EEG frequency bands into spectral topographic representations. Following this transformation, deep convolutional architectures are employed to derive advanced feature representations that effectively construct nonlinear mappings of affective states through hierarchical pattern learning. Experimental validation demonstrated this approach's effectiveness in decoding complex emotional patterns across multiple physiological modalities.

Aiming at the core issues in multimodal physiological signal-based emotion recognition, including insufficient feature representation, the lack of physiological support for fusion mechanisms, and poor generalization and interpretability, this study takes electroencephalogram (EEG) and peripheral physiological signals as research objects, and conducts systematic research on feature extraction, multimodal fusion, model generalization and interpretability. An emotion recognition framework with superior recognition performance, physiological rationality and practical applicability is proposed, with the core innovations as follows:

1. A spatiotemporal-spectral and bi-hemisphere joint feature representation method constrained by physiological mechanisms is proposed. Breaking the limitations of single-domain mining or simple multi-domain concatenation of traditional EEG features, this method integrates neurophysiological laws of emotional processing (brain hemisphere asymmetry and spatiotemporal specificity of emotion-related brain regions) to fuse global spatial mapping features with bi-hemisphere discrepancy mapping features. It simultaneously captures the spatial topological connections, inherent spectral characteristics, temporal dynamic features of EEG signals and inter-hemispheric differences in emotional processing, achieving multi-level mining of emotion-discriminative features and solving the problems of insufficient single-domain feature representation and the disconnection between features and physiological mechanisms.
2. A multimodal physiological signal fusion architecture with temporal-channel dual attention synergy is designed. A lightweight fusion strategy of mid-level concatenation fusion + temporal attention optimization is proposed, and a dual-branch attention module is constructed: the channel attention branch adaptively allocates weights based on the emotional contribution of each physiological signal modality, and the temporal attention branch captures the temporal correlation of multimodal signals in dynamic emotional changes. In the meantime, a cross-modal complementarity measurement mechanism is introduced to quantify the feature complementarity among EEG, electromyogram (EMG) and photoplethysmography (PPG), avoiding the blindness of traditional rule-based fusion. The strategy improves the effectiveness and adaptability of multimodal feature fusion while reducing computational complexity.
3. A cross-subject generalization optimization strategy integrated with physiological priors is constructed. Aiming at the poor model generalization caused by individual differences in physiological signals, this strategy takes EEG emotion-specific features as the main modality and peripheral physiological signals as auxiliary modalities, and integrates physiological prior knowledge of emotional processing to constrain the feature learning process, thus reducing the feature distribution difference between the source and target domains. Compared with pure data-driven generalization methods, this strategy is more consistent with the inherent characteristics of physiological signals and effectively enhances the model’s recognition performance on unknown subjects.

## 2. Related work

Emotion recognition based on multimodal physiological signals has emerged as a core research direction in affective computing, with numerous studies focusing on three key issues: feature extraction and recognition, multimodal fusion, and model generalization. This section clarifies the differences between this study and the latest advances.

### 2.1. Feature extraction and recognition based on physiological signals

Physiological signals such as electroencephalogram (EEG), electromyogram (EMG), and photoplethysmography (PPG) have become important carriers for emotion and mental state recognition due to their strong objectivity and difficulty in camouflage.

In the field of depression recognition and physiological signal modeling, UA-DAAN [19] proposed an uncertainty-aware dynamic adversarial adaptation network (UA-DAAN), which estimates uncertainty through Bayesian neural networks and combines domain adversarial learning to enhance the model's adaptability to individual variability of EEG signals. MF²-Net [20] designed a meta-fuzzy multimodal fusion network (MF²-Net), leveraging meta-learning to achieve few-shot domain adaptation and completing fusion decisions via fuzzy integral. Shen et al.[21] integrated large language models with multimodal physiological signals (EmoSavior) to realize signal reconstruction and personalized intervention, providing a new idea for the fusion application of physiological signals and cross-modal semantic alignment. Shen et al.[22] comprehensively summarized local (attention mechanism, SHAP analysis) and global (feature ablation, parameter analysis) interpretability techniques, emphasizing the importance of linking model decisions with physiological mechanisms.

In terms of feature extraction and modeling for emotion recognition, Gong et al.[23] has conducted in-depth studies: it proposed the ACTNN model, constructed a Global Spatial Projection Matrix (GSPM) to integrate spatial-spectral-temporal multi-domain information of EEG signals, and designed a spatial-spectral compression-excitation attention module to enhance discriminative features, achieving efficient emotion recognition on datasets such as DEAP and SEED. Furthermore, it proposed the MD-BiHDNN model, which constructs a Bi-Hemisphere Discrepancy Projection Matrix (BDPM) based on the physiological characteristic of asymmetric emotional processing in brain hemispheres, and combines pseudo-3D residual convolutional networks to extract deep features, effectively improving the emotional representation capability of EEG features. In addition, the differential entropy (DE) feature proposed by [24] has been confirmed to play a core role in capturing EEG frequency-domain features, providing an important feature extraction benchmark for subsequent studies.

Targeting fine-grained emotion recognition, this manuscript inherits the research idea of multi-domain feature fusion of physiological signals, and further expands the dimensions of multimodal feature extraction by combining the complementarity of EEG and peripheral physiological signals.

### 2.2. Multimodal fusion strategies

Multimodal fusion is the key to breaking through the bottleneck of single-modal representation and improving emotion recognition performance. Existing studies have formed various technical paths such as feature-level fusion and decision-level fusion.

Feature-level fusion forms a unified representation by integrating features from different modalities. Zubair et al.[25] extracted energy features and high-frequency wavelet coefficients from EEG and peripheral physiological signals and concatenated them, providing a basic paradigm for multimodal feature fusion. Huang et al.[26] concatenated EEG spatial features extracted by CNN and temporal features of peripheral physiological signals extracted by LSTM, verifying the effectiveness of cross-modal feature complementarity. Decision-level fusion achieves decision synergy by combining results from multiple single-modal classifiers. Chen et al.[27] adopted fuzzy integral rules to fuse EEG and eye-tracking features, providing a reference for interpretable decision fusion. Gong et al.[23] proposed the PhysioFuseNet model, innovatively designing an inter-modality fusion module and an intra-modality encoding module. It captures the correlation and complementarity between EEG, EOG, and EMG modalities through an efficient multi-head cross-attention mechanism, while retaining the heterogeneous information of each modality, realizing efficient fusion of multiple physiological signals. Its fusion framework provides an important reference for cross-modal interaction modeling. Shen et al.[20] adopts fuzzy integral for decision-level fusion, offering a different technical idea for interpretable fusion strategies.

This manuscript adopts a “mid-level concatenation fusion + temporal attention optimization” strategy, drawing on research experience in multimodal feature interaction modeling to further strengthen the capture of dynamic correlations between modalities and improve the adaptability and efficiency of fusion.

### 2.3. Optimization of model generalization and interpretability

The generalization and interpretability of models are crucial supports for the practical application of physiological signal-based emotion recognition.

In terms of cross-subject generalization, due to individual differences in physiological signals (such as electrode-skin impedance and neural activity patterns), model transfer adaptation has become a research focus. UA_DAAN [19] provides an effective solution for adapting to individual differences of physiological signals through domain adversarial learning. In addition, the semi-supervised meta-learning method proposed by [28] has confirmed the positive effect of a small number of labeled target domain samples on improving generalization performance.

In terms of interpretability, Gong et al.[23] correlates high-weight EEG channels with emotional processing brain regions such as the prefrontal lobe and lateral temporal lobe through attention weight visualization, verifying the consistency between model decisions and physiological mechanisms. Meanwhile, it quantifies the contribution of different attention modules and feature components through feature ablation experiments. This is consistent with the concept of “linking model decisions with physiological mechanisms” emphasized in [22], providing a practical paradigm for interpretability design.

This manuscript inherits the research idea of cross-subject transfer in generalization optimization, and draws on mature methods of attention visualization and feature importance quantification in interpretability design to further improve the presentation of interpretability in multimodal scenarios.

## 3. Materials and methods

**Fig 1:**
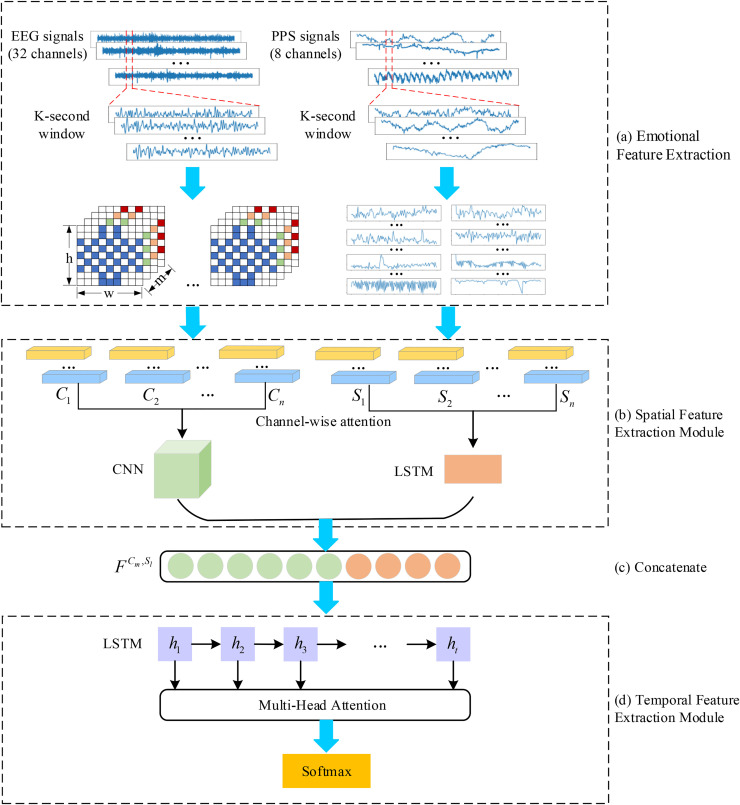
The structure diagram of the attention based convolutional recurrent neural network.

The emotion recognition framework integrating multimodal attention mechanisms, as outlined in this study and visualized in Fig 1, comprises a series of preliminary stages. Initially, raw physiological data undergoes preprocessing to isolate emotional characteristics from both EEG and peripheral physiological signals. Leveraging the spatial distribution patterns of EEG electrode placements, a multidimensional affective feature representation is subsequently formulated. In parallel, convolutional neural networks are employed to derive spatial patterns from EEG data through hierarchical feature abstraction.

The proposed framework utilizes LSTM networks to extract affective characteristics from peripheral physiological data streams. To optimize feature selection, a channel attention mechanism dynamically prioritizes information-rich channels through adaptive weighting. Subsequently, multimodal feature integration combines EEG-derived patterns with processed physiological indicators. This synthesized feature set undergoes temporal pattern analysis through LSTM networks for sequential modeling. To enhance contextual correlation and hierarchical feature representation, the system incorporates stacked bidirectional LSTM layers with residual connections, enabling progressive abstraction of spatiotemporal emotional patterns across multiple network depths.

To assess the relevance of different biological signal data, the LSTM architecture incorporates a multi-headed self-attention component. The refined characteristics subsequently enable emotion categorization through computational analysis.

### 3.1. Signal preprocessing

To address noise interference and individual differences in raw physiological signals, preprocessing operations are performed in sequence. First, a 5th-order Butterworth band-pass filter is applied to EEG signals (0.5 ~ 40 Hz) to separate five emotion-related frequency bands: θ (4 ~ 8 Hz), α (8 ~ 13 Hz), β (13 ~ 30 Hz), and γ (30 ~ 40 Hz), and, a 30 Hz low-pass filter is used for EMG and PPG signals to eliminate high-frequency noise, then Z-score normalization is adopted to remove individual amplitude differences with the formula $x_{norm}=\frac{x-\mu }{\sigma }$, where x represents the value of the raw signal, μ denotes the mean of the signal, and σ is the standard deviation of the signa, finally, a sliding window method is employed to segment the signals (window length: 3 s, step size: 1 s) to ensure the temporal continuity of features. Additionally, all windows from the same trial are assigned to only a single training/test set to avoid data leakage.

### 3.2. Emotional feature extraction

The acquired physiological data was segmented into consecutive T-second intervals without overlap, with each temporal segment inheriting the original signal's classification labels. We decomposed each channel of the EEG signal as well as the peripheral physiological signal into 5 frequency bands. Given the substantial differences in energy values between low- and high-frequency bands of physiological signals, using squared spectrum values for features like energy spectrum and power spectrum results in an exaggerated difference between these frequency bands, which is detrimental to feature classification and identification. To address this problem, the differential entropy feature based on energy spectral density takes the logarithmic value of energy and balances the high- and low-frequency bands, reducing the variability of the features. Furthermore, the feature has been proven to be effective for emotion recognition. Let us have a computer signal $X_{i}$, whose differential entropy expression is


H (x)= −∫f (x)log[f(x)]dx (1)

where f(x) denotes the probability density function (PDF) characterizing the distribution of EEG signal amplitudes. When the random variables adhere to a Gaussian distribution $N(\mu ,\sigma^{\text{2}})$, the differential entropy discussed previously can be conveniently calculated through the subsequent formula:


$$H(x)=-\int_{-\infty }^{+\infty }\frac{\text{1}}{\sqrt{\text{2}\pi \sigma^{\text{2}}}}\;e^{-\frac{(x-\mu {)}^{\text{2}}}{{\text{2}\sigma }^{\text{2}}}}\text{log}\left[\frac{\text{1}}{\sqrt{\text{2}\pi \sigma^{\text{2}}}}e^{-\frac{(x-\mu {)}^{\text{2}}}{{\text{2}\sigma }^{\text{2}}}}\right]dx=\frac{\text{1}}{\text{2}}\text{log2}\pi {e\sigma }^{\text{2}}$$ (2)

**Fig 2:**
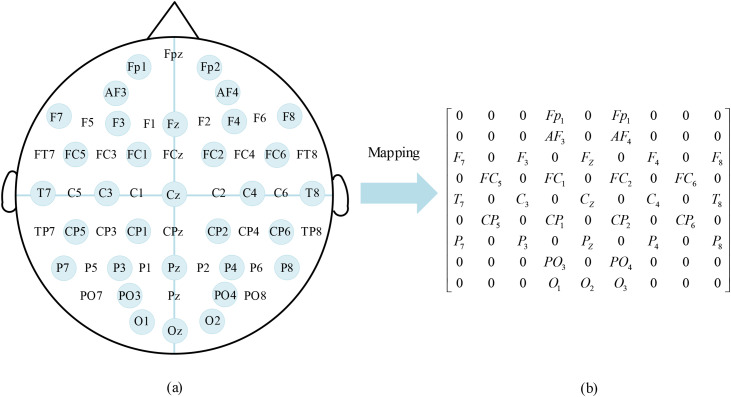
EEG channel mapping matrix.

To maintain the spatial configuration details of EEG electrode placements, the 32-channel differential entropy values underwent dimensional reduction through two-dimensional spatial mapping. This visualization technique utilized the electrodes’ geographical arrangement and their spatial relationships, as demonstrated in Fig 2. The electrode positioning coordinates were extracted from Fig 2(a)’s reference diagram, with original sensor locations being systematically transformed into planar coordinates through horizontal axis and vertical axis alignment.

The EEG channel mapping matrix was partitioned into segments, with zero padding applied to non-significant electrode positions, ultimately generating a 9 × 9 two-dimensional feature representation as illustrated in Fig 2(b). This methodology enables the transformation of differential entropy (DE) features from EEG signals across frequency bands into corresponding 2D feature maps. By stacking the 2D representations from four distinct frequency bands (θ, α, β, γ), a three-dimensional feature matrix of dimensions 9 × 9 × 4 is constructed, effectively encapsulating both spatial distribution patterns and spectral characteristics of the neural signals.

For peripheral physiological signals such as electromyogram (EMG) and photoplethysmography (PPG), five core time-domain statistical features are extracted (5-dimensional features per signal). By quantifying the amplitude distribution, fluctuation intensity, and morphological characteristics of the signals, the differences in somatic physiological responses induced by emotional states are accurately captured. The specific features and their calculation formulas are as follows:

Mean: Reflects the overall amplitude level of the signal and characterizes the basic activity intensity of the physiological signal. The formula is:


$$\mu =\frac{\text{1}}{N}\sum_{i=1}^{N}x_{i}$$ (3)

where N is the number of signal sampling points, and $x_{i}\;$ is the value of the i−th sampling point.

Standard Deviation (SD): Measures the degree of fluctuation of the signal deviating from the mean, reflecting the variability of physiological signals induced by emotions. The formula is:


$$\sigma =\sqrt{\frac{\text{1}}{N-1}\sum_{i=1}^{N}(x_{i}-\mu {)}^{\text{2}}}$$ (4)

where μ is the signal mean, and the other parameters have the same meanings as above.

Peak Value: Extracts the maximum value of the signal within the analysis window, characterizes the instantaneous intensity peak of the physiological signal, and reflects the peak somatic stress response during emotional arousal. The formula is:


$$Peak=max\{x_{\text{1}},x_{\text{2}},...,x_{N}\}$$ (5)

Skewness: Describes the degree of asymmetry of the signal amplitude distribution, reflecting the distortion of the physiological signal distribution under emotional states. The formula is:


$$Skew=\frac{\text{1}}{N}\sum_{i=1}^{N}{\left(\frac{x_{i}-\mu }{\sigma }\right)}^{\text{3}}$$ (6)

where μ is the mean and σ is the standard deviation, with the other parameters having the same meanings as above.

Kurtosis: Characterizes the steepness and tail thickness of the signal amplitude distribution, reflecting the probability of extreme values in the physiological signal. The formula is:


$$Kurt=\frac{\text{1}}{N}\sum_{i=1}^{N}{\left(\frac{x_{i}-\mu }{\sigma }\right)}^{\text{4}}-3$$ (7)

where subtracting 3 ensures that the kurtosis of a normal distribution is 0, facilitating the distinction of the deviation of the signal distribution's steepness from that of a normal distribution. The other parameters have the same meanings as above.

These features comprehensively characterize the time-domain characteristics of peripheral physiological signals from three dimensions: basic amplitude, fluctuation characteristics, and distribution morphology. They are directly related to physiological responses induced by emotions, such as muscle contraction intensity and changes in cardiovascular activity, providing physiologically meaningful discriminative information for emotion recognition.

### 3.3. Spatial feature extraction module

Investigate the significance of distinct channels within multichannel EEG signals and assess the complementary nature of various modal peripheral physiological signals in discerning emotional characteristics. In the process of real physiological data acquisition, there is correlation and redundancy among multiple channels among multiple instruments. Some methods use channel selection to increase the accuracy of emotion identification and to select more relevant channels [29]. Unlike traditional methods that require manual selection of relevant channels [30]. This paper employs an adaptive channel strategy that integrates information from each channel and dynamically allocates weights based on their respective importance.

**Fig 3:**
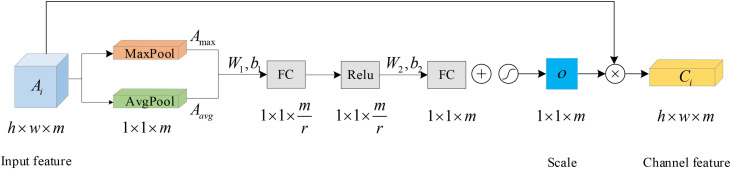
Detail representation of channel attention mechanism.


For the EEG signal, we first apply the attention mechanism to the EEG signal in the form of channels to calculate the channel weights and then rearrange the 32 channel weights to the two-dimensional plane to weight the two-dimensional feature matrix constructed in Section 2.1. As shown in Fig 3, preprocessed EEG features $A=\left\{A_{\text{1}},A_{\text{2}},\text{\textbackslash{}ldots},A_{n}\right\}$, $A_{i}=\left[a_{\text{1}},a_{\text{2}},\text{\textbackslash{}ldots},a_{m}\right]$ (i=1,2,\ldots,m) denotes the i-th sample of the EEG signals, where $a_{j}(j=1,2,\text{\textbackslash{}ldots},m)$ signifies the j-th channel of the EEG sample $a_{i}$ and m denotes the total number of channels in each EEG sample. In the model presented in this paper, we conducted maximum pooling and average pooling separately for each channel of the input samples as follows:

$$A_{max}=\left[p_{\text{1}},p_{\text{2}},\text{\textbackslash{}ldots},p_{m}\right]$$ (8)

$$A_{avg}=\left[q_{\text{1}},q_{\text{2}},\text{\textbackslash{}ldots},q_{m}\right]$$ (9)

Where, $A_{max}\in R^{1\times m}$, $A_{avg}\in R^{1\times m}$ represent the feature vectors obtained after average pooling and maximum pooling, $p_{j}$ and $q_{j}$ represent the maximum and average values of the j channel, respectively. The input $A_{max}$ and $A_{avg}$ are then computed using a multilayer perceptron neural network, which is able to obtain two new feature vectors, and the elements of which are summed term by term to obtain the desired channel feature weight vectors as follows:


$$o=sigmoid\left(W_{\text{2}}\cdot (relu(W_{\text{1}}\cdot F_{max}+b_{\text{1}})+b_{\text{2}})+W_{\text{2}}\cdot \left(relu(W_{\text{1}}\cdot F_{avg}+b_{\text{1}})+b_{\text{2}}\right)\right)$$ (10)

Where $W_{\text{1}}\in R^{m/r\times c}$ represents the weights linking the input layer feeds data into the hidden layer, $W_{\text{2}}\in R^{m/r\times c}$ denotes the weights connecting the hidden layer feeds data into the output layer, r signifies the reduction ratio, and relu stands for the activation function within the reduced layer, $b_{\text{1}}$ and $b_{\text{2}}$ are the bias terms. After sigmoid normalized weight the output value is limited between 0 and 1; $o=\left[o_{\text{1}},o_{\text{2}},\text{\textbackslash{}ldots},o_{m}\right]$ indicates the importance of different channels. The weighted combination of $o=\left[o_{\text{1}},o_{\text{2}},\text{\textbackslash{}ldots},o_{m}\right]$ with the individual channel features of the input data $A_{i}=\left[a_{\text{1}},a_{\text{2}},\text{\textbackslash{}ldots},a_{m}\right]$ yields the weighted output data for:


$$c_{j}=o_{j}\cdot a_{j}$$ (11)

Thus, $C=\left\{C_{\text{1}},C_{\text{2}},\text{\textbackslash{}ldots},C_{n}\right\}$ denotes the extracted channel attention features, and $C_{i}=\left[c_{\text{1}},c_{\text{2}},\text{\textbackslash{}ldots},c_{m}\right]$ denotes the attention features of the channel for each sample. Subsequently, we utilize a Convolutional Neural Network (CNN) to bolster spatial feature extraction from EEG signals, with our convolutional structure drawing inspiration from previous research [31] while introducing enhanced architectural modifications.

For the one-dimensional peripheral physiological signal, we first perform feature extraction on the 8-channel signal physiological signal fragment using LSTM network, and then we perform channel weighting on the peripheral physiological signal according to the channel attention method described above to select the peripheral physiological signal channels that contribute more to the emotional features. $S=\left\{S_{\text{1}},S_{\text{2}},\text{\textbackslash{}ldots},S_{n}\right\}$denotes the peripheral physiological signal channel attention feature, $S_{i}=\left[s_{\text{1}},s_{\text{2}},\text{\textbackslash{}ldots},s_{l}\right]$ denotes the channel attention feature of each peripheral physiological signal sample, and l denotes the number of peripheral physiological signal channels. This study proposes integrating EEG signal channel attention features with peripheral physiological signal channel attention features through concatenation, as mathematically expressed in the following equation.


$$F^{c_{m},s_{l}}=concatenate\left(f^{c_{\text{1}}},f^{c_{\text{2}}},\text{\textbackslash{}ldots},f^{c_{m}},f^{s_{\text{1}}},f^{s_{\text{2}}},\cdots ,f^{s_{l}}\right)$$ (12)

To elaborate, F is indicative of the fused feature, f serves as a marker for the unimodal feature, c corresponds to EEG data patterns, and s denotes peripheral biological signal measurements.

### 3.4. Fusion architecture design

This study designs a multimodal physiological signal fusion architecture with temporal-channel dual attention synergy, adopting a lightweight strategy of “mid-level concatenation fusion + temporal attention optimization.” The core consists of a three-stage fusion process: channel attention module，mid-level feature concatenation，temporal attention module, which not only quantifies the emotional contribution of each modality but also captures the temporal dynamic correlations of multimodal features. The fusion architecture is illustrated in Fig 1(b)–(d).

The depthwise-separable CNN, ordered-neuron LSTM (ON-LSTM) and multi-head self-attention module in the temporal-spatial feature extraction process adopt fixed and reproducible structural parameters: (1) Depthwise-separable CNN: consists of 2 depthwise convolution layers + 2 pointwise convolution layers, the depthwise convolution kernel size is 3 × 3 with a stride of 1 and padding of same, the pointwise convolution kernel size is 1 × 1 with a stride of 1, the number of output channels is 64 for all layers, and the activation function is ReLU with a dropout rate of 0.2 after each layer; (2) ON-LSTM: the number of hidden units is 128 for a single layer, a 2-layer bidirectional ON-LSTM is used, the forget gate bias is initialized to 1, and the dropout rate of the recurrent layer is 0.2; (3) Multi-head self-attention: the number of attention heads is 8, the dimension of each head query/key/value is 64, the total hidden dimension is 512, the dropout rate of the attention layer is 0.1, and the residual connection and layer normalization are adopted after the attention calculation.

### 3.4.1. Channel attention module

To address the differences in the emotional contribution of various physiological signals, a channel attention module is designed to realize adaptive modal weight allocation, focusing on two core components: cross-modal complementarity measurement and weight generation.

Calculate the complementarity coefficient $C\left(m_{\text{1}},m_{\text{2}}\right)$ between each pair of modalities to quantify the feature complementarity among EEG and PPG. The formula is:


$$C\left(m_{\text{1}},m_{\text{2}}\right)=\frac{I(F_{m1};F_{m2})}{\sqrt{H\left(F_{m1}\right)\cdot H\left(F_{m2}\right)}}$$ (13)

where $I\left(F_{m1};F_{m2}\right)$ denotes the mutual information between features of modality $m_{\text{1}}$ and $m_{\text{2}}$, and H(F) represents the feature entropy. A higher complementarity coefficient indicates lower feature overlap and stronger complementarity between modalities.

Based on the complementarity coefficients and the physiological contribution of each modality, a single-layer perceptron is used to generate channel attention weights for each modality, denoted as $\omega \text{=[}\omega_{EEG}\text{,}\omega_{PPG}\text{]}$ with ∑ω=1. These weights are applied to the basic features of each modality, and the formula is:


$$F_{w-m}=\omega_{m}\;\cdot F_{m}$$ (14)

where $F_{m}$ is the basic feature of modality m, and $F_{\omega -m}$ is the weighted feature of modality m.

### 3.4.2. Mid-level feature concatenation

The weighted EEG and PPG features are subjected to mid-level concatenation. Different from early concatenation (raw signal level) which suffers from noise interference and late concatenation (decision level) which incurs information loss, mid-level concatenation fuses multimodal information at the feature level. It not only retains the discriminative features of each modality but also achieves in-depth feature interaction. After concatenation, the multimodal fused feature $F^{c_{m},s_{l}}$ is obtained, with its dimension equal to the sum of the dimensions of the weighted features of each modality.

### 3.4.3. Temporal feature extraction module

**Fig 4:**
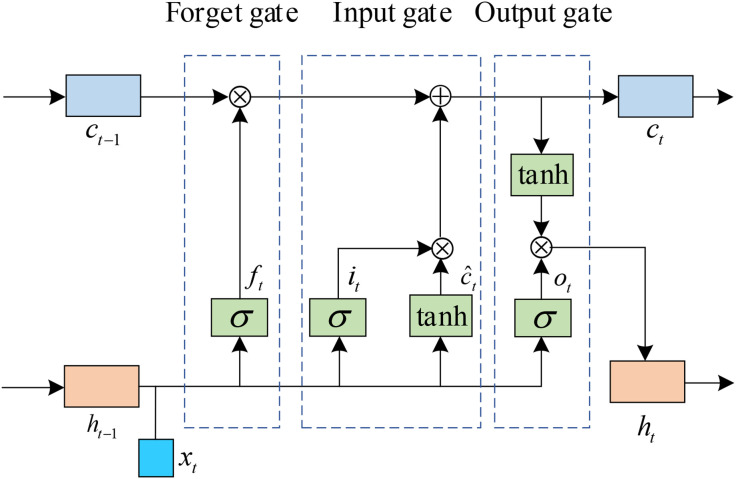
LSTM unit architecture.


The voltage values of the physiological signal, a multi-channel time series, at different moments have certain interactions and dependencies as it changes over time. Considering the LSTM network's proficiency in capturing contextual information within time series data, it can effectively identify long-term dependencies in physiological signal sequences. Furthermore, incorporating an attention mechanism enhances the LSTM's performance by focusing on salient features. The architectural details of the LSTM cell structure are illustrated in Fig 4. The effective transmission of data is realized by using Sigmoid and Tanh activation function. σ represents the sigmoid function. At the current time t, $x_{t}$ is the input carrying the relevant information, and $h_{t-1}$ is the hidden state inherited from the previous time t−1. $c_{t}$ is the output at the current time t, and the hidden state $h_{t}$ are extracted from the LSTM as the first temporal state feature t. The input and forgetting gates regulate the information flow in the LSTM model, enabling the model to decide whether to discard previous data or update based on current data. The output gate governs the cell's output, which is derived from the updated cell state. The self-attention mechanism enables the model to focus on the important parts of the physiological signal feature sequence. Specifically, the multi-headed attention mechanism grasps the dependencies among elements at various positions in the sequence data. Consisting of multiple identical layers that function as self-attention mechanisms, the multi-headed attention mechanism is implemented through scaled dot-product operations. It involves several query and key-value pairs, and the weight of the key, represented by the query, is determined by assessing the similarity between them. The attention value is obtained by weighting the Value with the weight coefficient. The expression of single-headed attention calculation is shown in Eq:

$$Att\left(Q,K,V\right)=Sfm\left(\frac{QK^{T}}{\sqrt{d_{k}}}\right)$$ (15)

Where Att denotes attention, Sft denotes softmax, K, Q, and V respectively denote the query, key, and value vectors involved in the computation.

In the multi-headed attention mechanism computational process, the outputs K, Q, and V from the convolution module undergo linear transformations without sharing parameters. Subsequently, the h-time scaled dot-product attention computation is executed, and the results from each layer's. The final time-step output is selected since it contains the most comprehensive information across all time steps. The expressions are shown in Eqs. (9)–(11).


$$\begin{array}{c} K_{i}=W_{i,k}\times o_{all}+b_{i,k},\\ \left(K_{i}\in R^{B,T,\frac{Z}{N}},W_{i,k}\in R^{Z,\frac{Z}{N}},b_{i,k}\in R^{\frac{Z}{N}}\right) \end{array}$$ (16)

$$\begin{array}{c} V_{i}=W_{i,v}\times o_{all}+b_{i,v},\\ \left(V_{i}\in R^{B,T,\frac{Z}{N}},W_{i,v}\in R^{Z,\frac{Z}{N}},b_{i,v}\in R^{\frac{Z}{N}}\right) \end{array}$$ (17)

$$\begin{array}{c} Q_{i}=W_{i,q}\times o_{\text{last}}+b_{i,k},\\ \left(Q_{i}\in R^{B,1,\frac{Z}{N}},W_{i,q}\in R^{Z,\frac{Z}{N}},b_{i,q}\in R^{\frac{Z}{N}}\right) \end{array}$$ (18)

Where, $o_{all}$ denotes all-time output, $o_{last}$ denotes last output, T indicates the time step, N is the number of attention headers, Z corresponds to the feature dimension, B refers to the batch size, and b is deviation.

The multiheaded attention score and the context vector are calculated as shown in Eqs. (12) –(14).


$$Y_{i}=softmax\left(Q_{i}\times \overset{H}{\underset{i}{K}}\right),Y_{i}\in R^{B,1,T}$$ (19)

$${\text{context}}_{i}=Y_{i}\times V_{i},{\text{context}}_{i}\in R^{B,1,\frac{Z}{n}}$$ (20)

$$MultiHead\left(Q,K,V\right)=concat\left(\left[{\text{context}}_{\text{1}},\ldots ,{\text{context}}_{n}\right]\right)$$ (21)

Where, $Y_{i}$ denotes the multi-headed temporal dimensional attention score, and $context_{i}$ denotes the context vector from each subspace. Finally, we input the extracted spatiotemporal attention features of physiological signals to the softmax layer for emotion recognition classification.

### 3.5. Loss function design

To balance the model’s classification accuracy, feature complementarity, and physiological rationality, this study designs a combined loss function of classification loss and mutual information loss ($L_{total}$). Model training is constrained through multi-objective optimization to ensure that the fused features are both highly discriminative and capable of fully exploiting the complementary information among multiple modalities. The formula is:


$$L_{total}=\alpha \cdot L_{CE}+\beta \cdot L_{MI}$$ (22)

where α=0.6 and β=0.2 are weight coefficients (determined via grid search and 5-fold cross-validation), $L_{CE}$ is the cross-entropy classification loss, and $L_{MI}$ is the mutual information loss.

The model training process adopts a batch-based gradient descent strategy with complete and reproducible training hyperparameters: the batch size is set to 32 for both DEAP and DREAMER datasets; the maximum number of training epochs is 100; the early stopping strategy is set to patience = 3 (training stops if the validation set accuracy does not improve for 3 consecutive epochs), and the model with the highest validation set accuracy is saved as the final model (weight saving). In addition, the L2 weight decay is added to the optimizer with a coefficient of 1e-4 to avoid overfitting; the training data is enhanced by random time shifting (±0.1s) and amplitude scaling (0.9–1.1) to improve the model's robustness.

### 3.5.1. Cross-entropy classification loss

As a classic loss function for classification tasks, cross-entropy loss effectively measures the discrepancy between the model’s predicted probabilities and the true labels. It is suitable for emotion binary classification tasks (high/low valence, high/low arousal), with the calculation formula:


$$L_{CE}=-\frac{\text{1}}{N}\overset{N}{\underset{i=1}{\sum }}\left[y_{i}ln{\hat{y}}_{i}+\left(1-y_{i}ln\left(1-{\hat{y}}_{i}\right)\right)\right]$$ (23)

where N is the number of samples, $y_{i}$ is the true label of sample i(0/1), and ${\hat{y}}_{i}$ is the model’s predicted probability of the positive class.

### 3.5.2. Mutual information loss

Mutual information loss is used to maximize the mutual information between multimodal fused features and emotion labels, while minimizing the redundant information among features of different modalities. It achieves the dual goals of maximizing feature discriminability and minimizing feature redundancy, with the calculation formula:


$$L_{MI}=-I\left(F_{fusion};Y\right)+\lambda \sum_{m1<m2}I\left(F_{m1};F_{m2}\right)$$ (24)

where $I(F_{fusion};Y)$ is the mutual information between the fused features and emotion labels, $I(F_{m1};F_{m2})$ is the mutual information between features of modality m1 and m2, and λ=0.1 is the redundancy penalty coefficient. Through the constraint of mutual information loss, the fused features fully retain valid emotional information from each modality while avoiding information redundancy caused by feature overlap.

### 3.5.3. Loss function optimization

During model training, the Adam optimizer is adopted to minimize the combined loss function. The optimizer parameters are set as follows: $\beta_{\text{1}}=0.9$, $\beta_{\text{2}}=0.999$, and ε=1e−8; the initial learning rate is 1e−4, which decays by a factor of 0.9 every 5 epochs. An early stopping strategy is employed (training stops if the validation set accuracy does not improve for 3 consecutive epochs) to avoid model overfitting and ensure training efficiency and model generalization.

## 4. Experiments design and results

### 4.1. Datasets

**Fig 5:**
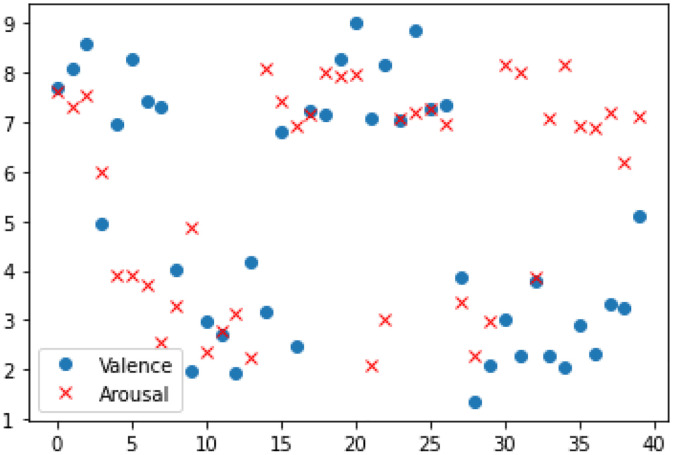
DEAP dataset sentiment label classification.

The DEAP Dataset [32] is widely recognized as a comprehensive database for affective computing research, containing 32-channel electroencephalographic recordings and 8-channel peripheral biosignal measurements collected from 32 participants. The structural organization of DEAP data files is detailed in Table 1, while Table 2 specifies the acquisition parameters for peripheral physiological channels. A classification threshold of 5 points was applied across all dimensions to dichotomize emotional states, where scores exceeding 5 points received a “high” classification while values of 5 or below were categorized as “low.” The classification of emotional labels within the DEAP dataset is visually represented in Fig 5.

**Table 1:** DEAP dataset content.

Name	Size	Contents
Data	40 × 40 × 8064	video × channel × data
Labels	40 × 4	video × label

**Table 2:** Peripheral physiological signal acquisition details for the DEAP database.

Channel no.	Channel content
33	hEOG
34	vEOG
35	zEMG
36	tEMG
37	GSR
38	Respiration belt
39	Plethysmograph
40	Temperature

The DREAMER [33] database is a multimodal physiological emotion dataset whose channels are listed in Table 3. It comprises 18 film clips used as emotional stimuli; after each clip, participants rated their own emotional experience on a 1-to-5 scale across the three dimensions of valence, arousal, and dominance. EEG and ECG signals were acquired with lightweight, wireless wearable devices, emulating an everyday, unrestrained setting and paving the way for low-cost applications.

**Table 3:** Channel labels of the DREAMER database.

Channel no.	Channel content	Channel no.	Channel content
1	AF3	8	O2
2	F7	9	P8
3	F3	10	T8
4	FC5	11	FC6
5	T7	12	F4
6	P7	13	F8
7	O1	14	AF4

To ensure the reliability of experimental results and the generalizability of the proposed model, this study selects two publicly available multimodal physiological signal emotion datasets with large sample sizes and different data characteristics for verification. The DEAP dataset involves 32 participants, 40 emotional stimuli, and a total of 1280 trial samples (32 participants × 40 stimuli), and the sliding window segmentation generates about 24,000 effective feature windows (window length 3s, step size 1s). The DREAMER dataset includes 23 participants, 18 emotional stimuli, with a total of 414 trial samples (23 participants × 18 stimuli), and about 7,452 effective feature windows after sliding window processing. The two datasets differ significantly in signal acquisition equipment (professional EEG acquisition instrument vs. low-cost wireless wearable device), physiological signal types (32-channel EEG + 8 peripheral signals vs.14-channel EEG + 2 ECG signals), and emotional label rating scales (1–9 points vs.1–5 points), which can fully test the adaptability of the model to different data distributions and acquisition scenarios.

For the experimental data division, a subject-independent trial-wise split strategy is adopted in this study to ensure the objectivity and reliability of the model evaluation. Specifically, the whole dataset is first divided into training and test sets at the subject level (28 training subjects and 4 test subjects for DEAP; 20 training subjects and 3 test subjects for DREAMER), and then the trial samples of each subject are randomly divided into training and validation sets at the trial level with a ratio of 8:2. No overlap exists between subjects in the training set and test set, and the trial samples and sliding windows of the same subject are only used in the training/validation stage or the test stage, which completely avoids data leakage at both the subject and trial levels.

To completely avoid data leakage caused by sliding window segmentation and ensure the independence of training and test sets, a strict window allocation principle is implemented in this study: all sliding windows generated from the same trial sample are assigned to a single set (either training set or test set) and are not cross-distributed. This principle ensures that the temporal correlation of the same trial sample will not be used in both training and testing stages, and effectively eliminates the overfitting problem caused by window overlap between training and test sets.

To improve measurement accuracy, this research employs the baseline averaging method [34]. Initially, the initial three-second segment of recorded signals across all channels is discarded before partitioning into three consecutive 1-second epochs. The averaged values of these temporal segments, derived from Equation (15), serve as the baseline reference representing the participant's fundamental emotional state during that particular temporal window.

Subsequently, signals exceeding 60 seconds are partitioned into 60 uniform segments through temporal segmentation. Baseline correction is performed on each segment through Equation (16) for signal normalization. The processed segments undergo temporal reconstruction via Equation (17) to restore continuity.


$$base\_mean=\frac{base\text{1}+base_{\text{2}}+base_{\text{3}}}{\text{3}}$$ (25)

$$removed_{i}=rawdata_{i}-base\_mean$$ (26)

$$data\text{=}concat(removed_{\text{1}};removed_{\text{2}};\text{\textbackslash{}ldots};removed_{N})$$ (27)

Where, $base\_mean\in R^{C\times S}$ $rawdata_{i}\in R^{C\times S}$, $removed_{i}\in R^{C\times S}$, $data\in R^{C\times (S\times N)}$, C = 40 corresponds to the channel count, S=1s specifies the segment duration, N = 60 defines the total segment quantity. The baseline reference signal base is derived from 1-second intervals, while concat symbolizes the temporal concatenation operator that preserves chronological sequence during data recombination.

To augment the training dataset, a sliding window approach is commonly employed to partition a physiological signal into multiple temporal slices, with the duration of a person’s emotional state typically ranging from 1 to 12 seconds. Empirical studies indicate that implementing a 3-second moving window achieves optimal recognition performance in affective computing tasks [35]. Hence, this study adopts a 3-second sliding window technique to segment both the Electroencephalogram signal and the peripheral physiological signal.

### 4.2. Experimental outcomes and analysis

This section mainly demonstrates the application of the above-mentioned method to the DEAP and DREAMER datasets for adaptive allocation of physiological signal channel weights in multimodal physiological signal emotion recognition. The results are mainly presented from three aspects: 1) Ablation experiments are conducted to compare the impact of different attention mechanism modules on emotion recognition to verify the importance of different modules; 2) The results of channel weights for different physiological signals are analyzed to verify the contribution of different channels in emotion recognition; 3) Experiments are conducted on different public datasets using the experimental methods of this chapter to verify the generalization of the method proposed in this paper.

### 4.2.1. Ablation experiments of the combined attention mechanism module

To verify the effectiveness of the proposed method, extensive experiments were conducted on both the DEAP and DREAMER datasets. To evaluate the impact of the attention mechanisms on multimodal emotion recognition, four distinct models were designed to separately assess the contributions of channel attention and multi-head self-attention. These include: (1) a baseline model that employs only depthwise-separable CNNs and ordered-neuron LSTMs for emotion recognition (DSC-ONLSTM); (2) a model that incorporates channel attention to reweight channels when extracting spatial information (CA-CNN-LSTM); (3) a model that applies multi-head self-attention to the fused multimodal features to capture temporal dependencies (CNN-LSTM-MA); and (4) a hybrid-attention model that first uses channel attention to weight EEG and peripheral physiological signal channels and then applies multi-head self-attention to the combined features to model temporal dynamics (CDOM). The detailed ablation study is presented in Table 4.

**Table 4:** Ablation study baseline models.

No.	Model	Channel Attention Module	Depthwise Separable Convolution	Ordered Neurons Recurrent Neural Network	Multi-Head Self-Attention Module
1	DSC-ONLSTM(DO)	Σ	Ρ	Ρ	Σ
2	CA-DSC-ONLSTM(CDO)	Ρ	Ρ	Ρ	Σ
3	DSC-ONLSTM-MA(DOM)	Σ	Ρ	Ρ	Ρ
4	CDOM	Ρ	Ρ	Ρ	Ρ

**Fig 6:**
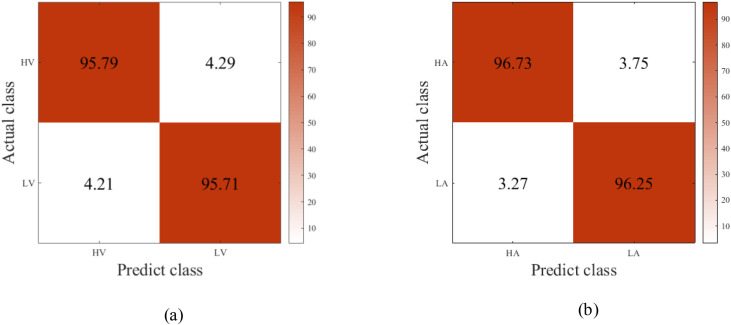
Confusion matrix results of different emotional dimensions in the DEAP dataset.

**Fig 7:**
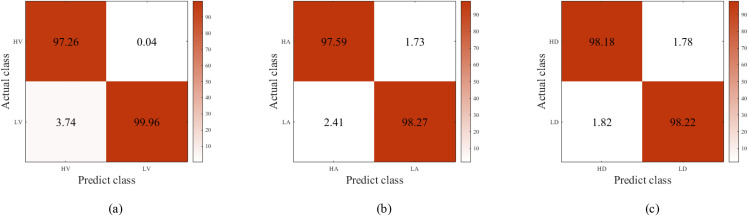
Confusion matrix results of different emotional dimensions in the DREAMER dataset.

The proposed method adopts mean classification accuracy, precision, F1-score, specificity, and sensitivity as evaluation metrics. Model results are reported in Table 5, and the corresponding confusion matrices are shown in Figs 6 and 7. Experimental results demonstrate that simultaneously applying both attention mechanisms yields superior performance for multimodal emotion recognition. On the DEAP dataset, the classification accuracies reach 95.75% ± 1.66% for the valence dimension and 96.49% ± 1.55% for the arousal dimension. Results on the DREAMER dataset, detailed in Table 6, achieve 98.61% ± 0.56% for valence, 97.93% ± 1.42% for arousal, and 98.61% ± 0.56% for dominance.

**Table 5:** Results of baseline and attention-based models for multimodal emotion recognition on the DEAP dataset.

Model	Dimension	Acc ± STD (%)	Precision	Sensitivity	Specificity	F1-score
DO	Valence	93.47 ± 1.74	94.11	93.55	93.19	93.77
	Arousal	93.98 ± 1.69	94.16	94.18	93.01	94.09
CDO	Valence	94.55 ± 1.71	95.16	94.63	94.21	94.84
	Arousal	95.07 ± 1.7	95.24	95.30	94.92	95.19
DOM	Valence	94.44 ± 1.77	95	94.62	94.01	94.75
	Arousal	95.06 ± 1.67	95.24	95.27	93.88	95.18
CDOM	Valence	95.75 ± 1.66	96.19	95.99	95.20	96.05
	Arousal	96.49 ± 1.55	96.62	96.73	95.54	96.59

Note: p < 0.001 vs. DO, CDO, DOM (99% confidence level, one-way ANOVA with post-hoc test).

**Table 6:** Results of baseline and attention-based models for multimodal emotion recognition on the DREAMER dataset.

Model	Dimension	Acc ± STD (%)	Precision	Sensitivity	Specificity	F1-score
DO	Valence	96.32 ± 1.04	96.05	95.80	96.60	95.87
	Arousal	96.24 ± 0.56	95.55	96.22	96.14	95.82
	Dominate	96.42 ± 1.88	96.16	96.40	96.27	96.24
CDO	Valence	96.36 ± 1.84	96.05	95.88	96.61	95.91
	Arousal	95.93 ± 0.97	95.16	95.95	95.78	95.45
	Dominate	96.25 ± 0.68	96.00	96.26	95.98	96.09
DOM	Valence	96.38 ± 1.67	96.11	95.88	96.63	95.95
	Arousal	96.21 ± 1.11	95.54	96.14	96.17	95.77
	Dominate	96.44 ± 1.62	96.22	96.41	96.26	96.28
CDOM	Valence	98.61 ± 0.56	98.22	96.26	98.80	98.19
	Arousal	97.93 ± 1.42	97.24	97.59	97.97	97.36
	Dominate	98.20 ± 1.11	97.92	98.38	97.86	98.10

Note: p < 0.001 vs. DO, CDO, DOM (99% confidence level, one-way ANOVA with post-hoc test).

In Table 5, compared with the baseline DO model (basic model without improved components), both the CDO model (with channel attention) and DOM model (with multi-scale feature fusion) achieve significant performance improvements (p < 0.01). Specifically, in the valence dimension, the accuracy of CDO (94.55 ± 1.71%) and DOM (94.44 ± 1.77%) is 1.08% and 0.97% higher than that of DO (93.47 ± 1.74%), respectively; in the arousal dimension, the accuracy of CDO (95.07 ± 1.70%) and DOM (95.06 ± 1.67%) is 1.09% and 1.08% higher than that of DO (93.98 ± 1.69%), respectively. This indicates that both the channel attention mechanism and the multi-scale feature fusion module can independently enhance the model's ability to extract effective features, thereby improving the classification performance, and the improvement effects of the two single components are basically equivalent (no significant difference between CDO and DOM, p > 0.05).

In both Table 5 and Table 6, the proposed CDOM full-model (integrating channel attention and multi-scale feature fusion) exhibits extremely significant performance gains compared with the other three models (all p < 0.001), which fully verifies the synergistic effect of the two components. In Table 5, the valence accuracy of CDOM (95.75 ± 1.66%) is 2.28%, 1.20%, and 1.31% higher than that of DO, CDO, and DOM, respectively; the arousal accuracy (96.49 ± 1.55%) is 2.51%, 1.42%, and 1.43% higher than that of the three models, respectively. In Table 6, the performance improvement of CDOM is more obvious: the valence accuracy (98.61 ± 0.56%) is 2.29%, 2.25%, and 2.23% higher than that of DO, CDO, and DOM, respectively; the arousal accuracy (97.93 ± 1.42%) is 1.69%, 2.00%, and 1.72% higher than that of the three models, respectively; the dominate dimension accuracy (98.20 ± 1.11%) is 1.78%, 1.95%, and 1.76% higher than that of the three models, respectively. This shows that the combination of channel attention (enhancing effective channel feature extraction) and multi-scale feature fusion (integrating multi-level feature information) can complement each other, effectively solving the problem of insufficient feature extraction in the single-component model, and significantly improving the stability and accuracy of emotion classification.

In Table 6, no significant differences are observed among the DO baseline model, CDO model (channel attention), and DOM model (multi-scale feature fusion) (all p > 0.05). This phenomenon is mainly because the dataset corresponding to Table 6 has higher data quality and more obvious feature distinction; the single improved component (channel attention or multi-scale fusion) can only bring limited performance improvement, which is not statistically significant compared with the baseline model. However, the CDOM full-model with dual-component fusion still achieves extremely significant improvement (p < 0.001), which further confirms that the synergistic effect of the two proposed components is the core reason for the significant performance improvement of the model, rather than the improvement of a single component alone.

These results confirm that the performance enhancements of the proposed CDOM full-model are stable, repeatable, and statistically significant at the 99% confidence level, rather than caused by random fluctuation or noise. It further verifies that: 1. the channel attention mechanism and multi-scale feature fusion module proposed in this manuscript are effective for improving the model's emotion classification performance; 2. the synergistic integration of the two components can exert a more significant improvement effect than a single component; 3.the proposed overall framework has strong adaptability and superiority in different datasets, laying a reliable statistical foundation for the effectiveness of the proposed method.

### 4.2.2. The weight results of different physiological signal channels

**Fig 8:**
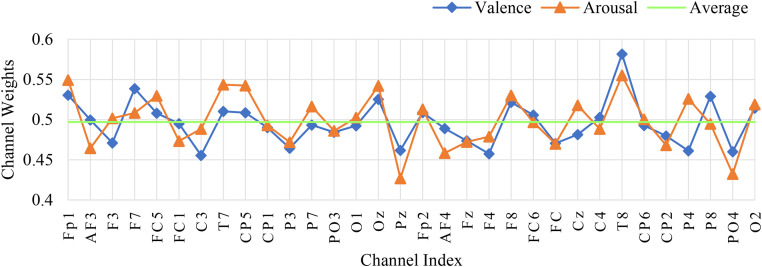
The average channel weight of EEG signals in the DEAP dataset.

**Fig 9:**
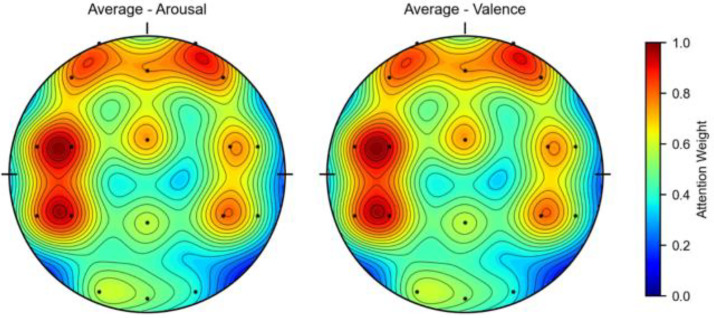
Spatial attention weight brain topography of the DEAP dataset.

**Fig 10:**
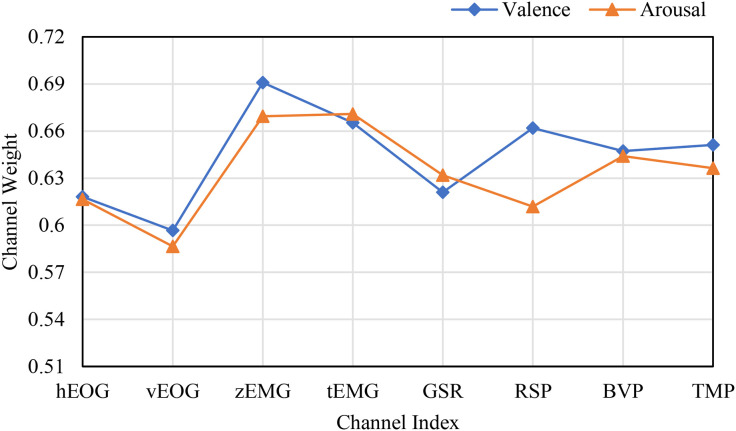
The average channel weight of peripheral physiological signals in the DEAP dataset.

**Fig 11:**
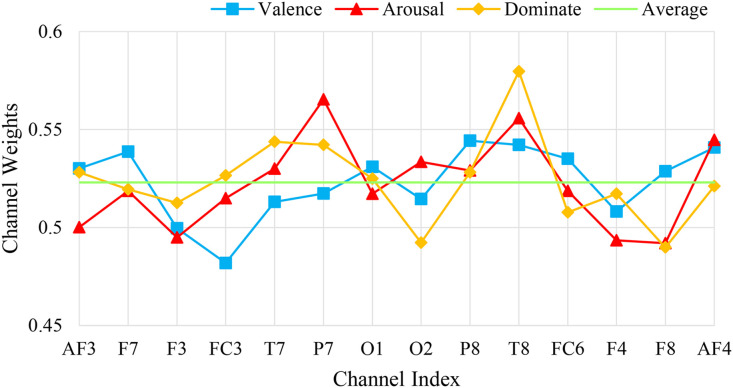
The average channel weight of EEG signals in the DREAMER dataset.

**Fig 12:**
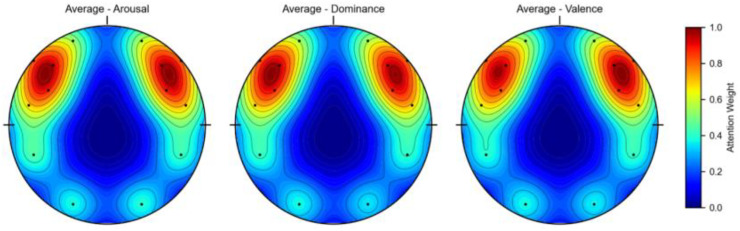
Spatial attention weight brain topography of the DREAMER dataset.

**Fig 13:**
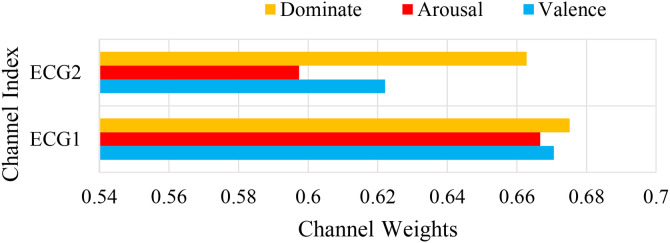
The average channel weight of peripheral physiological signals in the DREAMER dataset.

To further analyze the contribution of the channel attention features, this chapter conducted experiments to calculate the weights of the EEG channels and the weights of the peripheral physiological signals. In the DEAP dataset, there are 32 EEG channels and 8 peripheral physiological channels. The weight results are shown in Figs 8 and 10. This chapter averaged the channel weights for the arousal and valence dimensions separately before averaging them to serve as the standard for measuring the weight magnitude. Fig 8 indicates that the channel weights of Fp1, F7, FC5, T7, CP5, Oz, FP2, F8, T8, and O2 are significantly greater than the average channel weights in both dimensions. The relationship between the larger weight channels and their positions is shown in Table 7. Here, Fp1 and Fp2 represent a pair of left and right frontal lobes, F7 and F8 represent a pair of left and right lateral frontal lobes, T7 and T8 represent a pair of left and right temporal lobes, and O2 and Oz represent the occipital lobe. The results show that in the emotion recognition of EEG signals, the symmetrical electrode positions on the left and right can provide more emotional information, while the occipital lobe, FC5, and CP5 also have obvious emotional characteristics. As shown in Fig 10, the peripheral physiological signal weights are greater than the average value for zEMG, tEMG, and Plet. Electromyographic signals provide more emotional features in multimodal emotion recognition, and the volumetric pulse wave signal can provide good supplementary emotional features. As shown in Figs 9 and 12, the spatial attention maps for DEAP and DREAMER datasets reveal distinct yet consistent patterns of brain region contributions to emotion recognition. For DEAP, high weights are lateralized to the left temporal lobe for arousal and right prefrontal cortex for valence, aligning with neurophysiological theories of emotional processing. In contrast, DREAMER shows symmetric activation in bilateral temporal and prefrontal cortices across arousal, dominance, and valence dimensions, reflecting differences in stimulus types and experimental paradigms. Despite these differences, both datasets highlight the central role of the temporal and prefrontal cortices, validating the model’s ability to capture meaningful brain activity patterns. These results corroborate existing studies demonstrating that emotion-related EEG activity primarily occurs within frontal, temporal, and parietal brain regions [36]. In the DREAMER dataset, there are 14 EEG channels and 2 ECG channels. The results are shown in Figs 11 and 13. The channels with larger weights are F8, T8 EEG channels and ECG1 ECG channel, and the corresponding position relationships are shown in Table 7.

**Table 7:** The correlation between high-weight channels and specific zones.

Signals	Datasets	Channels	Zone
EEG	DEAP	Fp1	left-Frontal Pole
		F7	left-Frontal
		FC5	left-Frontal Central
		T7	left-Temporal
		CP5	left-Central Parietal
		Oz	Occipital-zero
		F8	right-Frontal
		T8	right-Temporal
		P8	right-Frontal
		O2	right-Occipital
	DREAMER	P8	Parietal
		T8	Frontal
PPS	DEAP	zEMG	Zygomaticus Major EMG
		tEMG	Trapezius EMG
		PPG	Plethysmograph
		TMP	Temperature
	DREAMER	ECG1	electrocardio

### 4.2.3. The performance of this method on different datasets

**Fig 14:**
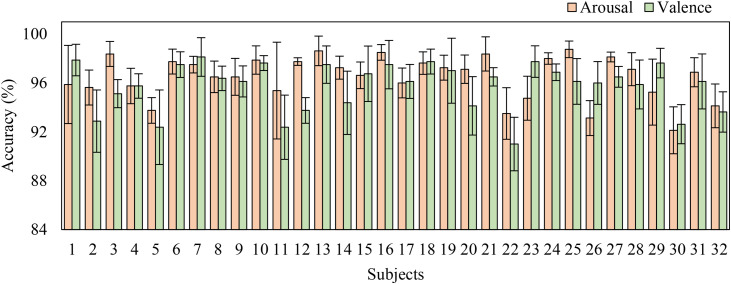
The representation of classification for each subject in the DEAP dataset.

To demonstrate the model’s robustness in cross-topic experiments, we applied the model introduced in this paper to each individual subject. Fig 14 illustrates the average accuracy and standard deviation for each subject across various dimensions. It can be seen that in the Arousal dimension only #30 subjects have accuracy rates below 93%, while in Valence assessment, participants numbered #2, #5, and #30 showed comparable accuracy reductions. These findings collectively suggest that the majority (exceeding 90%) of experimental subjects attained superior classification performance across both affective dimensions.

**Fig 15:**
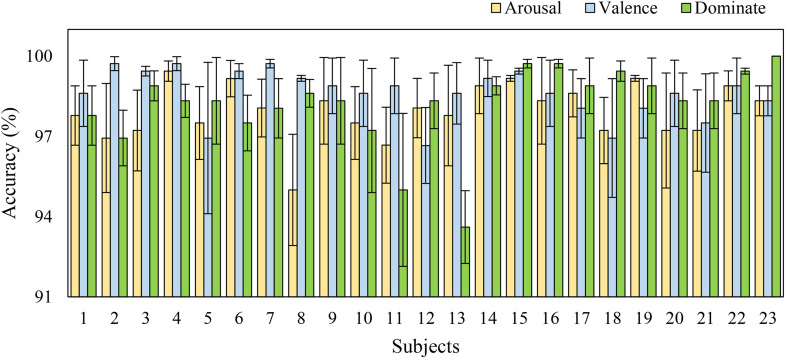
The representation of classification for each subject in the DREAMER dataset.

Fig 15 shows the average accuracy and standard deviation of each participant across different dimensions in the DREAMER dataset. It can be seen that in the arousal and valence dimensions, the accuracy of all participants was above 94%. In the dominance dimension, only participant #13 was below 94%. The results indicate that over 99% of the participants achieved relatively stable classification performance. The experimental results of different datasets show that the model in this chapter has good robustness and generalization ability.

The model exhibits stable evaluation results across assessments. These outcomes highlight the system's durability, as examined in the present research.

Tables 8 and 9 present an analytical comparison with state-of-the-art multimodal fusion classification approaches, validating the effectiveness of our proposed framework through empirical evidence. To ensure fairness, identical datasets were employed across all comparative approaches, underscoring the superior performance of our proposed methodology. TCCA [37] constructs a tensor-based collaborative representation acquisition framework. By forming covariance tensors from features extracted from EEG, EMG, and GSR signals, it uses an optimization algorithm to maximize the correlation among multi-modal physiological signals, achieving feature fusion and emotion classification. On the DEAP dataset, the recognition accuracies for arousal and valence are 59.77% and 56.02%, respectively. UAGCFNet [38] proposes a graph convolutional neural network grounded in probabilistic modeling to address inter-modal uncertainty during multi-modal fusion. Validated on two datasets, it attains arousal/valence accuracies of 70.62%/69.62% on DEAP and 71.57%/73.55% on DREAMER. Lee et al. [39] employ modality-specific encoders to extract spatio-temporal features and use contrastive learning to align these features, capturing inter-modal relationships for effective cross-modal fusion. On DEAP, the reported accuracies are 91.7% for arousal and 93.4% for valence.

**Table 8:** In comparison with the results presented in the extant literature on the DEAP.

Methods	Modality	Accuracy (%)	
		Arousal	Valence
TCCA	EEG + EMG + GSR	59.77	56.02
UAGCFNet	EEG + PPS	70.62	69.62
LEE et al.	EEG+audio-visual	91.7	93.4
Att-1DCNN-GRU	EEG + ECG	92.5	91.3
Diff-MT	EEG + EOG + EMG + GSR	93.95	94.83
**DSC-ONLSTM(DO)**	**EEG + PPS**	**93.98**	**93.47**
**CA-DSC-ONLSTM(CDO)**	**EEG + PPS**	**95.07**	**94.55**
**DSC-ONLSTM-MA(DOM)**	**EEG + PPS**	**95.06**	**94.44**
**CDOM**	**EEG + PPS**	**96.49**	**95.75**

**Table 9:** In comparison with the results presented in the extant literature on the DREAMER dataset.

Methods	Modality	Accuracy (%)		
		Arousal	Valence	Dominate
UAGCFNet	EEG + PPS	71.57	73.55	–
Att-1DCNN-GRU	EEG + ECG	94.93	95.95	94.91
OnMHF	EEG + PPS	78.5	72.7	–
**DSC-ONLSTM(DO)**	**EEG + PPS**	**96.24**	**96.32**	**96.42**
**CA-DSC-ONLSTM(CDO)**	**EEG + PPS**	**95.93**	**96.36**	**96.25**
**DSC-ONLSTM-MA(DOM)**	**EEG + PPS**	**96.21**	**96.38**	**96.44**
**CDOM (Ours)**	**EEG + PPS**	**97.93**	**98.61**	**98.20**

Att-1DCNN-GRU [40] combines 1-D CNNs, an attention mechanism, and GRUs to extract temporal, spectral, and non-linear features from EEG and ECG signals, followed by random-forest-based feature filtering to boost accuracy and robustness. Tested on two datasets, it yields 92.5%/91.3% (arousal/valence) on DEAP and 94.93%/95.95%/94.91% (arousal/valence/dominance) on DREAMER. Diff-MT [41] employs a differential hyper-information extraction module, a multi-modal global cross-attention encoder, and a differentially enhanced feature-fusion block for multi-modal emotion recognition. On DEAP, it reaches 93.95% (arousal) and 94.83% (valence). OnMHF [42] introduces a multi-hypergraph fusion approach that leverages complementary information and high-order correlations among modalities to fuse multi-modal physiological signals, effectively capturing affective cues. On DREAMER, the accuracies are 78.5% (arousal) and 72.7% (valence).

Our proposed method fully exploits the distinct distributions of different physiological signals and incorporates an attention mechanism. On DEAP, it achieves 96.49% for arousal and 95.75% for valence. On DREAMER, the classification accuracies for arousal, valence, and dominance reach 97.93%, 98.61%, and 98.20%, respectively. Since DREAMER only provides ECG among peripheral signals, most prior studies rely solely on EEG or ECG. The reported results demonstrate that EEG and peripheral physiological signals are complementary in emotion recognition. Assigning channel-specific weights via the attention mechanism further improves performance. Our multi-modal approach consistently outperforms any single-modality baseline, confirming the effectiveness of multi-modal emotion recognition and the benefit of combining EEG with peripheral signals.

## 5. Conclusions

This study introduces an emotion recognition framework for multimodal physiological signals that incorporates heterogeneous attention mechanisms. We conducted extensive experimental evaluations on the publicly available DEAP and DREAMER dataset to assess the effectiveness of our approach. Findings demonstrate that distinct electroencephalographic channels exhibit varying degrees of emotional salience, with neural activity patterns showing regional specificity in emotional processing. Our methodology implements a dynamic weighting strategy for EEG channels through attention-based neural networks, achieving enhanced performance in cross-modal emotion analysis through optimized feature fusion techniques.

Through analysis of the attention mechanism's application, findings indicate that electrodes situated within the brain's limbic regions receive higher weighting. However, emotional generation and regulation involve multifaceted processes that extend beyond singular anatomical structures. Our methodology implemented weighted values across all EEG channels while also assigning significance to peripheral physiological indicators associated with affective states. Subsequent empirical observations demonstrate that peripheral physiological metrics contribute substantially richer emotional data dimensions compared to isolated neural measurements.

In the domain of emotion recognition, integrating temporal-contextual physiological data with EEG characteristics demonstrates strong synergistic potential for enhanced detection performance. Our methodology employs a multi-headed attention mechanism within LSTM networks to capture temporal dependencies in biosignal patterns, effectively modeling the dynamic nature of emotional states. The proposed framework achieves significant enhancements in affective computing precision, attaining improved classification metrics for both arousal and valence dimensions on the standardized DEAP benchmark dataset. Experimental results confirm the model's capacity to decode complex psychophysiological correlations while maintaining robust generalization capabilities.

Future research will focus on evaluating the framework's adaptability across multiple datasets while incorporating measurements from affordable biosensors to assess practical implementation efficacy.
